# Hyperons in neutron stars: studies of hyperon spectroscopy and the hyperon–nucleon interaction with the K-long Facility

**DOI:** 10.1098/rsta.2023.0124

**Published:** 2024-07-23

**Authors:** N. Zachariou, S. Fegan, D. Watts, M. Bashkanov

**Affiliations:** ^1^ School of Physics Engineering and Technology, University of York, York YO10 5DD, UK

**Keywords:** hyperons, neutron stars, hadron spectroscopy

## Abstract

Elucidating the role of strange baryons (hyperons) in neutron stars requires detailed knowledge of hyperon–nucleon interactions in the light (u,d,s) quark sector. The structure of the hyperons and their excitation spectra also directly impact, and are an input to, models of big-bang nucleosynthesis. The upcoming K-long Facility will provide a much-needed intense and clean neutral strange meson beam, from which hyperons can be produced at rates where hyperon structure, hyperon–nucleon interactions and higher-order interactions can be studied with a new level of accuracy and for hitherto unreachable measurements. The new facility has the potential to address long-standing questions surrounding the strange sector of the strong force and its relevance to the structure of atomic nuclei, neutron stars and the cosmos at large.

This article is part of the theme issue ‘The liminal position of Nuclear Physics: from hadrons to neutron stars’.

## 1. Introduction

Neutron stars are one of the most extreme objects in the universe, being remnants of massive stars that have undergone supernova explosions at the end of their lives. These incredibly dense stars have masses between 1.4 and 3 times that of the Sun with radii only of about 5–10 km [1], resulting in central densities in the range of 4–8 times the normal nuclear matter saturation density [2]. The true nature of neutron stars remains an open question. Establishing the equation of state (EoS) of neutron stars to describe the relationship between various physical properties of the matter inside the neutron star is an active field of research [3].

Traditionally, the core of neutron stars has been modelled as a uniform fluid of neutron-rich nuclear matter in equilibrium with respect to the weak interaction [4]. However, owing to the extreme conditions, it is more energetically favourable for new hadronic degrees of freedom to appear. Heavy neutron stars are predicted to have significant hyperon fractions in their cores, which significantly soften the EoS and influence basic properties, such as the maximum mass and cooling mechanisms. This large contribution from hyperons is owing to their ability to condense (owing to their quantum mechanical distinguishability from nucleons), and the strong Pauli blocking of hyperon decays in the medium of the star. Despite their importance, the predicted effect of the hyperons varies significantly with different models of the hyperon–nucleon interaction.

This is currently reflected in the so-called ‘hyperon puzzle’ [5], which is a long-standing issue in nuclear and particle physics, with theoretical predictions of neutron stars with hyperon matter resulting in maximum masses that are currently inconsistent with recent astronomical observations. The hyperon puzzle highlights the tension between theoretical models of dense nuclear matter, which often predict the presence of hyperons, and observational constraints on neutron star masses, which imply that hyperons might not be present in the cores of neutron stars or that their effects are somehow mitigated.

Addressing the hyperon puzzle lies in obtaining a comprehensive picture of the strong interaction, which can be accessed by introducing the strangeness degree of freedom in the, now well-understood, nucleon–nucleon (NN) interaction. The NN interaction has a long history of detailed studies, reflected by the high-precision measurements currently available [6], with phenomenological approaches being able to describe observed phenomena with high accuracy. On the other hand, the interaction between hyperons and nucleons (YN) is very poorly constrained, mainly owing to difficulties associated with performing high-precision scattering experiments involving these short-lived hyperons.

The experimental history of studying the hyperon–nucleon interaction spans several decades, which started soon after the discovery of the first hyperon (ground state Λ ) in the early 1950s [7], and involves various experimental techniques aimed at understanding the properties of hyperons and their interactions with nucleons. Early attempts in the 1960s and 1970s focused on experiments using bubble chambers and provided the current database available for the two-body interaction between hyperons and nucleons, with only about 1300 scattering events observed [6]. Later efforts shifted to assessing the hyperon–nucleon interaction through detailed studies of hypernuclear physics, which investigated hyperons bound in atomic nuclei as the observed hypernuclear energy levels and decays are sensitive to the YN interaction. Although giving valuable input to the YN interaction, such studies have significant model uncertainties related to medium modification and many-body effects. For example, the available models reproduce the basic hypernuclear features but fail to describe details of the effective two-body interactions in hypernuclei. Therefore, alternative and more direct approaches—with different and smaller systematic uncertainties—are needed to adequately constrain and understand the YN interaction in detail.

Here we present current efforts in underpinning the hyperon–nucleon interaction using a novel approach that enables access to the exclusive two-body (and higher-order three-body) scattering process with high statistics. We also discuss the experimental programme currently underway at Thomas Jefferson Laboratory that gives constraints on the nature of the strong interaction through their influence on the excitation spectra of strange hadrons. The excitation spectra are also critical input to models of big-bang nucleosynthesis with the majority of visible matter in the universe progressing through excited hyperon states following condensation from the quark–gluon plasma [8].

## 2. Hyperon–nucleon interaction

A novel approach has recently been employed that gives clean access to the two-body and three-body interaction between (free) hyperons and nucleons. This uses the photoproduction of energy and momentum-tagged hyperons and studying their subsequent interaction within the same target cell. This is the cleanest method for producing a secondary hyperon beam. There are no complicating effects from initial-state interactions of the beam particles (photons), and the electromagnetic production processes typically produce smaller beam-related background events in the detectors than for strongly interacting beams (e.g. pion or kaon beams). Furthermore, the incident photon beam can be polarized, enabling the produced hyperon beam to also be polarized [9,10], a truly unique and powerful capability. Tagged hyperon beams give access to exclusive scattering processes on free nucleons, mitigating against the complicating model-dependent uncertainties inherent in many-body effects (e.g. hypernuclear studies). Stringent constraints on the YN interaction also provide much-needed input to the hypernuclear studies and offer a complementary route to extracting the many-body effects, particularly the three-body hyperon–nucleon interaction, which are critical to solving the ‘hyperon puzzle’ [5]. (We note that such three-body processes will also be studied at Jefferson Lab using hyperon scattering from deuterium targets.)

**Figure 1.**
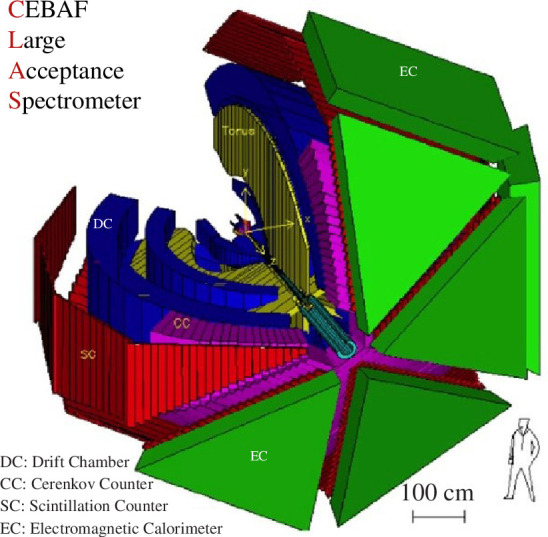
The diagrammatic representation of CLAS, which is operated in Jefferson Lab Hall B from 1997 until 2012.

Analysis of hyperon photoproduction experiments carried out using the CEBAF large-acceptance spectrometer (CLAS; see figure 1) at Thomas Jefferson Laboratory with a high-intensity energy tagged photon beam enabled the first high-precision measurement of the elastic scattering of hyperon–proton ( Λ –p) scattering [11]. This study achieved the first statistically precise measurement of this two-body interaction, significantly enhancing the world database over the sparse bubble chamber experiments. Large-acceptance detectors, such as CLAS, appear crucial for achieving such measurements both in terms of statistical accuracy and the understanding and removal of inelastic scattering events. Linearly and circularly polarized photon beams, in combination with the self-analyzing (spin-dependent) decays of hyperons, give access to a large set of polarization observables that are crucial for constraining the dynamics of the YN interaction. Deuteron targets will give constraints on hyperon–deuteron scattering and hyperon-induced disintegration ( Λd→Λd and Λd→Λpn ). A wealth of new data can be expected in the coming years with studies extended to Σ hyperon interactions with nucleons ( Σn→Σn and Σp→Σp ).

However, these studies possible with photon beams at Jefferson Lab are not enough. Hyperon–nucleon scattering and structure deeper into the strangeness sector are required but are not feasible from (zero-strangeness) photon beams interacting in the target cell. This need will be addressed using strange meson beams produced from a new K-long Facility (KLF) [12], where the more favourable production vertex in the target cell means Ω and cascade hyperons can be produced with sufficient rates to accurately determine their scattering for the first time. The scattering datasets for lighter hyperons will also be improved very significantly. The intense neutral kaon beam will be produced from new compact photon source (CPS) technologies [13]. As discussed in §4, the CPS is used for the production of an intense photon beam, which is then impinged on a beryllium target located downstream to produce a high flux of neutral kaons. The CPS beam intensities will reach up to five orders of magnitude beyond that currently achievable, opening up a wealth of new hadron and nuclear physics perspectives for a future experimental programme. This facility, which is expected to go online in 2026, will elucidate the deeper strange quark sector of hadron physics with unprecedented precision.

**Figure 2.**
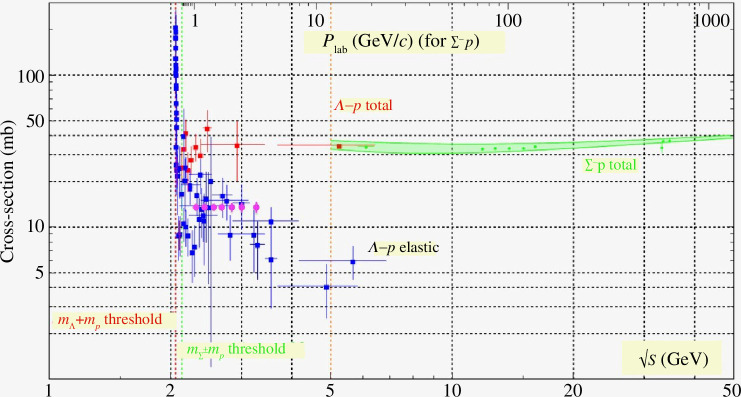
Projected measurements at the KLF of elastic cross-sections for Λ –*p* up to 4 GeV in hyperon momentum (magenta), compared to world data. Figure adapted from Particle Data Group (PDG) [6].

With this new KLF, studies on the hyperon–nucleon interaction will focus on obtaining a dataset in which a hyperon beam, produced on a nucleon and tagged by the detection of the pion ( $K_{L}p\to \pi^{+}\Lambda /\Sigma$ ) or kaon in ( $K_{L}p\to K\Xi$ ), rescatters with a secondary nucleon within the long target cell. The large-acceptance GlueX detector will enable the reconstruction of the event by the detection of the hyperon decay products. The KLF, which facilitates a copious production of hyperons, will provide us with unprecedented statistics to study the YN interaction, for the single strange hyperons and access for the first time to study the interaction of doubly strange baryons with nucleons ( ΞN interaction). Figure 2 illustrates the expected statistical precision for measurements of the Λ *–p* elastic cross-section as compared to the current world database. Using the self-analyticity of hyperons allows us to obtain further stringent constraints on the underlying dynamics and address the ‘hyperon puzzle’. The KLF will allow a much more precise measurement than the recently obtained measurement from CLAS, as it will provide ∼ 40 times higher statistics and a significant increase in kinematical coverage.

## 3. Strange hyperon spectroscopy

By improving knowledge of the fundamental hyperon–nucleon interactions, the programme will also provide a wealth of information on hyperon excitation spectra, from single- to triple-strangeness systems. Such information, currently sparse or missing, provides constraint on fundamental non-perturbative Quantum ChromoDynamics (QCD) in the strange sector, as predicted from Lattice [14], ξ EFT and quark model [15] methodologies. In fact, many more states are predicted than currently observed in the experiment. In cosmology, $\Xi^{*}$ and $\Omega^{*}$ spectra strongly influence the mechanisms of hadronic freeze out from the QGP in the μ s-old universe [8]. As only the two lowest $\Xi^{*}$ and the ground state Ω are established [6], cosmologists resort to model predictions and uncertain states with uncertain $J^{P}$ . The close relationship between the (unknown) excitation spectra and the evolution of the early universe creates a ‘compelling experimental program’ [8].

The Hall D KLF measurements will allow studies of very poorly known multiplets of $\Lambda^{*}$ , $\Sigma^{*}$ , $\Xi^{*}$ and even $\Omega^{*}$ hyperons with unprecedented statistical precision. These measurements also have the potential to observe dozens of predicted (but heretofore unobserved) states and to establish the quantum numbers of already observed hyperon resonances listed in PDG2022 [6]. Interesting puzzles exist for Particle Data Group (PDG)-listed excited hyperons that do not fit into any of the low-lying excited multiplets, and these need to be further revisited and investigated. Excited Ξ s, for instance, are very poorly known. Establishing and discovering new states is important, in particular, for the determination of the multiplet structure of excited baryons.

Specifically, the experiment will measure both differential cross-sections and self-analyzed polarizations of the produced Λ , Σ , Ξ and Ω hyperons with measurements spanning centre-of-mass cos⁡θ from −0.95 to 0.95 in the range *W* = 1490–2500 MeV. The new data will significantly constrain the partial wave analyses; reduce model-dependent uncertainties in the extraction of the properties and pole positions of the strange hyperon resonances; and establish the orbitally excited multiplets in the spectra of the Ξ and Ω hyperons. Comparison with the corresponding multiplets in the spectra of the charm and bottom hyperons will provide insight into the accuracy of QCD-based calculations over a large range of masses.

Determination of the $\Omega^{*}$ excitation spectrum, via $K^{0}p\to K^{+}K^{+}\Omega -$ , provides a fundamental challenge to QCD theory. Specifically, Baryons with symmetric quark content offer lucid QCD interpretation. Analogous excitation spectra are predicted for symmetric baryons across the quark sectors ( Ω (sss), Ω (ccc) and Ω (bbb)), but strange sector quark dynamics mean Ω (sss) has larger splitting between states [16]. A powerful new constraint on our understanding of baryon dynamics, effectively hidden for heavy sectors, is realisable while providing access to the strangeness 1 baryon sector for complementary measurements from the ones currently underway in Hall B at Thomas Jefferson Laboratory [17].

As well as excited hyperon states, the $d^{*}$ has the potential for tremendous impact in hadron and astrophysics [18–20]. Initial observation of this exotic hadron was made in 2011 [21,22], and it has the potential to play a pivotal role in hadron and neutron star physics if confirmed as a compact hexaquark. Theoretical work on the potential of $d^{*}$ bosonic condensates contributing to neutron stars and even dark matter [23], motivated γ -astronomy analyses [24–26] and discussed for neutron star gravitational lensing observations, e.g [27]. Specifically, the $d^{*}$ provides a possible new phase transition for compressed hadronic matter in neutron stars and softens the path to quark matter transitions. Its inclusion in the EoS for neutron stars produces a mass limit in agreement with that recently extracted from gravitational waves observation of neutron stars near the limits of stability [18–20]. Experiments at Thomas Jefferson Laboratory provide the necessary tools and framework that enables a detailed study of such exotic states.

Furthermore, the discovery of heavier strange $d^{*}$ states ( $d_{s}^{*}$ , $d_{s\,s}^{*}$ and $d_{s\,s\,s}^{*}$ ) [28] in the predicted SU(3) decuplet would be further evidence of its hexaquark origin and cement its role in hadron and astrophysics with all the impacts discussed previously. Such states could potentially play a role in the dynamics of strange matter analogous to the $d^{*}$ for light quark matter (e.g. strange stars [29]). At KLF, the $d_{s\,s}^{*}$ will become accessible through a simple charge exchange reaction on the deuteron.

## 4. Description of the KLF

**Figure 3.**
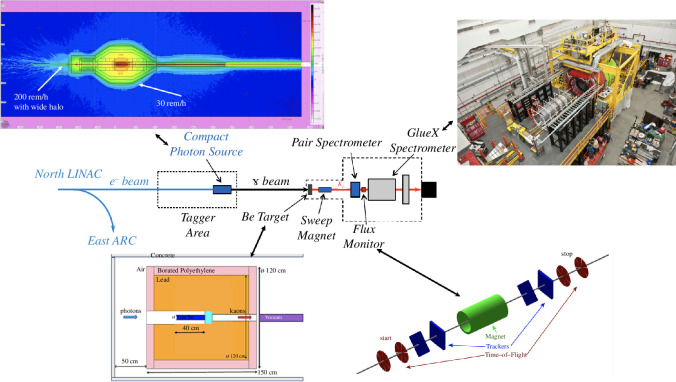
Artist’s impression of Jefferson Lab Hall D configured for the KLF, showing the Kaon Flux Monitor positioned upstream of the GlueX spectrometer, the beryllium target and the CPS.

The KLF project will establish a secondary *K*_*L*_ beamline at Jefferson Lab Hall D (see figure 3) for scattering experiments on both proton and neutron targets in order to determine a large set of observables using the large-acceptance GlueX detector [30]. The GlueX spectrometer in Hall D at Jefferson Lab is a large-acceptance detector employed by the GlueX Collaboration to investigate a wide range of topics in meson and baryon spectroscopy and structure, particularly the search for mesons with excited gluonic content, using the recently upgraded 12 GeV electron beam of CEBAF accelerator [31]. The spectrometer is carefully designed to measure the complete electromagnetic response for nucleons and nuclei across the kinematic plane: elastic, resonance, quasi-elastic and deep inelastic reactions with almost 4 π acceptance for all final state particles.

The secondary neutral kaon beam will be generated by directing a high-energy, high-intensity photon beam from a CPS [13] (producing six orders of magnitude larger γ flux than currently employed at Thomas Jefferson Lab) onto a beryllium target upstream of the GlueX detector. The flux of the $K_{L}$ beam is expected to be $∼1\times 10^{4}\;K_{L}/s$ on a liquid hydrogen/deuterium cryogenic target within the recently upgraded GlueX detector. This flux will allow statistics in the case of the hydrogen target to exceed that of earlier experiments by almost three orders of magnitude.

The ability of the GlueX spectrometer to measure reaction fragments over wide ranges in polar θ and azimuthal ϕ angles with good coverage for both charged and neutral particles, together with the KL energy information from the KL time-of-flight, provides an ideal environment for studies of hyperon spectroscopy and the hyperon–nucleon interaction.

## 5. Summary

Through a dedicated programme at Thomas Jefferson Lab, the coming decade will see a step change in our understanding of hyperons, in both their interactions with nucleons and their underlying structure. Such information will be crucial for our understanding and testing of QCD in the strange sector and for progressing our understanding of compact nuclear matter as found in neutron stars. The KLF at Thomas Jefferson Lab is instrumental in the investigation of the hyperon–nucleon interaction and hyperon spectroscopy, enabling precision measurements of Λ and Ξ interaction with nucleons and a comprehensive investigation of the hyperon excited states.

## Data Availability

This article has no additional data.
